# The long-noncoding RNA SOCS2-AS1 suppresses endometrial cancer progression by regulating AURKA degradation

**DOI:** 10.1038/s41419-021-03595-x

**Published:** 2021-04-06

**Authors:** Fangfang Jian, Xiaoxia Che, Jingjing Zhang, Chang Liu, Gedan Liu, Yujing Tang, Weiwei Feng

**Affiliations:** 1 Department of obstetrics and gynecology, Ruijin Hospital, Shanghai Jiao Tong University School of Medicine, Shanghai, 200025 China; 2 Obstetrics and gynecology hospital, Fudan University, Shanghai, 200011 China

**Keywords:** Non-coding RNAs, Endometrial cancer

## Abstract

Aberrant long-noncoding RNA (lncRNA) expression has been shown to be involved in the pathogenesis of endometrial cancer (EC). Herein, we report a novel tumor suppressor lncRNA SOCS2-AS1 in EC. Quantitative real-time PCR was performed to detect RNA expression. In situ hybridization and nuclear/cytoplasmic fractionation assays were used to detect the subcellular location. We found that SOCS2-AS1 was downregulated in EC tissues. Its reduced expression was correlated with advanced clinical stage and poor prognosis. Forced expression of SOCS2-AS1 suppressed EC cell proliferation and induced cell-cycle arrest and apoptosis. SOCS2-AS1-binding proteins were detected using RNA pull-down assay and mass spectrometry. Mechanistically, SOCS2-AS1 bound to Aurora kinase A (AURKA) and increased its degradation through the ubiquitin-proteasome pathway. In conclusion, SOCS2-AS1 may thus serve as a prognostic predictor and a biomarker for AURKA-inhibitor treatment in EC patients.

## Introduction

Endometrial cancer (EC) is the most frequently occurring gynecologic cancer in developed countries—with over 319,000 cases diagnosed worldwide, and in excess of 76,000 deaths annually^1^. Most women diagnosed with EC have early-stage disease and show favorable outcomes, which is particularly true for the well-differentiated, endometrioid histologic subtype^2^. However, there is a subset of low-grade, early-stage, well-differentiated endometrioid tumors in which unexpected recurrences and poor outcomes do occur^3^. Clinical outcomes worsen considerably for women with recurrent or advanced disease, and for women diagnosed with a clinically aggressive histologic subtype such as the serous histotype^4^. Indeed, although patient outcomes for most cancers have improved over the past 2 decades, mortality rates for uterine cancer have increased >20%^5^. Thus, there is an urgent need to develop more effective strategies for the diagnosis and treatment of this cancer.

Long-noncoding RNAs (lncRNA) constitute a large class of non-protein-coding transcripts that are over 200 bases in length^6^. Mounting evidence shows that many lncRNAs are aberrantly expressed in a broad spectrum of cancers, and that they play key roles in promoting and maintaining cancer characteristics^7,8^. Currently, only a few lncRNAs have been shown to be involved in EC—including MEG3^9^, HOTAIR^10^, MEAT1^11^, MALAT1^12^, BANCR^13^, H19^14^, and LINC00672^15^.

To identify novel EC-implicated lncRNAs, we herein performed RNA-seq to analyze lncRNA expression profiles in EC. We identified a collection of novel candidate EC-implicated lncRNAs that are relevant to diverse biologic processes, and identified a novel tumor-suppressive lncRNA, SOCS2-AS1, with crucial biologic and clinical impact on EC. We demonstrated that SOCS2-AS1 inhibited EC cell proliferation and tumorigenicity in vivo and in vitro, and that low expression levels of SOCS2-AS1 predicted a poor prognosis in EC patients. Mechanistically, SOCS2-AS1 exerts its tumor-suppressive activity via promotion of Aurora kinase A (AURKA) degradation. SOCS2-AS1 may thus serve as a new indicator for EC treatment and prognosis.

## Materials and methods

### Clinical specimens

Twenty-one EC samples (3 with clear cell carcinoma, 5 with serous carcinoma, and 13 with endometrioid endometrial carcinoma) and five normal samples (from endometria at the proliferative stage) from the Obstetrics and Gynecology Hospital, Fudan University were used for RNA sequencing. Another cohort consisting of 19 normal endometrial and 47 EC tissues obtained from the Obstetrics & Gynecology Hospital of Fudan University was used for subsequent quantitative polymerase chain reaction (qPCR) validation. We also developed tissue microarrays containing 72 paired formalin-fixed and paraffin-embedded specimens (of endometrial carcinoma and paracarcinoma tissues) that represented different stages of EC in patients who underwent surgical resection at Ruijin Hospital affiliated to Shanghai Jiaotong University School of Medicine between 2015 and 2019. This present study was approved by the Ethics Committee of Shanghai Jiao Tong University School of Medicine.

### Reagents and cell lines

Human EC cell lines (Ishikawa, HEC-1A, KLE, ECC-1, AN3-CA, and RL95-2) were purchased from ATCC and maintained in Dulbecco’s Modified Eagle Medium (DMEM) /F12 (Gibco, Carlsbad, CA, USA) and RPMI-1640 medium (Gibco) containing 10% fetal bovine serum (Gibco) and 100 U/mL of penicillin/streptomycin in a humidified incubator of 5% CO_2_ in compressed air at 37 °C. We purchased all reagents from Sigma-Aldrich unless stated otherwise.

### Quantitative real-time PCR (qPCR)

Total RNA was extracted with TRIzol reagent (Invitrogen, Carlsbad, CA), and cytoplasmic and nuclear RNA was separated and purified using the PARIS Kit (Life Technologies, Carlsbad, CA, USA) according to the manufacturer’s instructions. For reverse transcription, 1 μg of total RNA was converted to cDNA in a 20-μl reaction volume using a reverse transcription kit (Promega) following the manufacturer’s instructions, and qPCR was performed on a LightCycler 480 system (Roche) using SYBR Green Supermix (Takara). The relative RNA levels were calculated on the basis of 2 ^ΔCT^ and normalized to the housekeeping gene glyceraldehyde 3-phosphate dehydrogenase (GAPDH) or 18 S ribosomal RNA. We executed quantification in quadruplicate, and the experiments were repeated independently three times. Primer sequences are listed in Supplementary Table 1.

### Western immunoblotting analysis

Total cell lysate was prepared with radioimmunoprecipitation assay (RIPA) buffer containing Protease Inhibitor Cocktail, and we measured protein concentrations by bicinchoninic acid protein assay reagent (Bio-Rad) and resolved extracts using sodium dodecyl sulfate polyacrylamide gel electrophoresis on 10% gels. Membranes were blocked for 2 h at room temperature in TBS-Tween-20 containing 5% nonfat dried milk (Bio-Rad), washed, and then incubated with primary antibodies at 4 °C overnight. The following primary antibodies were used: anti-AURKA (1:1000, Cell Signaling Technology), anti-caspase-3 (1:1000, Cell Signaling Technology), anti-PARP (1:1000, Cell Signaling Technology), anti-cleaved-caspase-3 (1:1000, Cell Signaling Technology), anti-cleaved-PARP (1:1000, Cell Signaling Technology), anti-pH2AX (1:1000, Cell Signaling Technology), anti-SOCS2 (1:1000, Cell Signaling Technology), anti-flag (1:1000, Cell Signaling Technology), and anti-GAPDH (1:1000, Cell Signaling Technology). After washing, membranes were incubated with horseradish peroxidase-conjugated secondary antibodies (Cell Signaling Technology), and resulting signal was detected using enhanced chemiluminescence (PerkinElmer, Waltham, MA, USA).

### Plasmids and stable cell lines

The siRNA transfections were conducted with the lipofectamine 3000 (Thermofishier scientific) according to the manufacturer’s instructions. The target sequences of AURKA siRNAs (GenePharma) were as follows: 1, 5′-GCACAAUUCUCGUGGCUACUUUCACUU-3′; 2, 5′-CUCUAUAAACUGUUCCAAGUGGUGCAU-3′. The SOCS2-AS1 sequence was cloned into the lentiviral vector PGLV3/H1 (GenePharma) for stable expression in EC cells. The shRNA sequences against SOCS2-AS1 (1, GGACTTCTCAATACAGGAGCC; 2, AGAACAAAGGCAACAGAGAAG; 3, CCTGACACCTCACTCTAAATC) were cloned into PGLV2/H1(GenePharma). The shRNA sequences against AURKA (1, GGACCTGTTAAGGCTACAGCT; 2, ACCTGTAAATAGTGGCCAGGC) were cloned into PGLV3/H1 (GenePharma). Stable cell lines were generated through transduction with packaged lentivirus. Transduced cells were selected for stably infected cells with puromycin (1 μg/ml).

### RNA immunoprecipitation (RIP) assays

RIP experiments were performed using the Magna RIPTM RNA-Binding Protein Immunoprecipitation Kit (Millipore, MA, USA) according to the manufacturer’s instructions. Cells were washed using pre-cooled phosphate-buffered saline (PBS), lysed using RIPA lysis in an ice bath for 1 hour, and centrifuged at 13,000 × *g* for 10 min at 4 °C to collect the supernatant. We subsequently incubated the supernatant with an antibody for coprecipitation. Specifically, magnetic beads from each coprecipitation system were washed, resuspended in RIP wash buffer, and incubated separately with antibodies—including AURKA and IgG (1:100; Abcam, ab109489). Next, the bead-antibody complex was rinsed, resuspended in RIP Wash Buffer, and incubated with cellular extract at 4 °C overnight. The samples were then placed on the bead pedestal to collect the complex of bead protein. RNAs were eluted and extracted with Trizol reagent (Invitrogen) and quantified by qPCR. To demonstrate that the detected RNA signals specifically bound to AURKA, total RNA (input controls) and normal rabbit IgG controls were assayed simultaneously.

CCK8, RTCA, flow cytometry, and RNA sequencing were performed as described previously^16^.

### RNA pull-down assay and mass spectrometry (LC-MS/MS) analysis

Full-length sense and antisense SOCS2-AS1 strands were cloned into a pGEM-T Easy Vector (Promega). In vitro transcription was performed using the MEGAscript^TM^ T7 transcription kit (Cat.AM1333, Thermo), and RNA was purified using the RNeasy MinElute Cleanup Kit (QIAGEN). We labeled SOCS2-AS1 using the Pierce^TM^ RNA 3′-end desthiobiotinylation kit (Cat. 20163, Thermo Fisher Scientific). RNA pull-down assays were accomplished using a Pierce Magnetic RNA-Protein Pull-Down Kit according to the manufacturer’s instructions (Cat. 20164, Thermo). Cells were harvested and resuspended in freshly prepared radioimmunoprecipitation assay lysis buffer supplemented with RNaseOUT recombinant ribonuclease inhibitor (Thermo Fisher Scientific), SUPERase In RNase inhibitor (Thermo Fisher Scientific), and a protease/phosphatase inhibitor cocktail (Roche). In brief, 20 mg of total biotin-labeled RNA was first restructured in RNA structure buffer at 90 °C for 2 min, immediately placed on ice for 2 min, and then incubated at room temperature for 20 min. Labeled RNA was incubated with streptavidin magnetic beads (Pierce) for 30 min at room temperature with agitation, and the RNA-captured magnetic beads were washed twice with wash buffer (Thermo Fisher Scientific). Sense and antisense RNAs captured on the magnetic beads (as well as blank beads) were incubated with cell lysates in protein-RNA-binding buffer (Thermo Fisher Scientific) overnight at 4 °C with agitation. RNA-binding protein complexes were washed five times with ice-cold wash buffer and boiled in SDS lysis buffer. We analyzed the retrieved proteins by tandem mass spectrometry (Beijing Protein Innovation, Beijing, China) or western immunoblotting. LC-MS/MS experiments were performed with an LTQ linear ion trap mass spectrometer (Thermo Finnigan, San Jose, CA) equipped with a microspray source.

### Fluorescence in situ hybridization (FISH)

SOCS2-AS1, U6, and 18S FISH probes were synthesized by RiboBio (Guangzhou, China), and FISH was conducted using the FISH kit according to the manufacturer’s protocol (RN: 10910; RiboBio). The cells immobilized by 4% polyoxymethylene were incubated with permeabilizing solution (0.5% Triton X-100 diluted in PBS) at 4 °C for 5 min. After washing three times with PBS, the cells were treated with pre-hybridization buffer at 37 °C for 30 min. Then, 20 μM probe mix that was diluted in hybridization buffer was incubated overnight at 37 °C after removing pre-hybridization buffer. The DNA was stained with 4',6-diamidino-2-phenylindole (DAPI) dye for 10 min before sealing, and we observed images with a confocal microscope (Leica, Solms, Germany). We also performed FISH on EC tissue microarrays and rated staining intensity as 0 (absent), 1 (weak), 2 (moderate), or 3 (intense). The percentage of staining was also rated as 0 (0%), 1 (1–25%), 2 (26–50%), 3 (51–75%), or 4 (76–100%). We calculated the immunohistochemistry score (IHS) by multiplying the quantity and intensity scores, and scores ranged from 0 to 12. An IHS score of 0–6 was low, and 7–12 showed high immunoreactivity.

### Single-cell electrophoresis assay (Comet assay)

Single-cell gel electrophoresis (Neutral) was done according to the manufacturer’s instructions. In brief, indicated cells were collected, followed by washing and resuspension in ice-cold PBS, and 1–2 × 10^4^ cells were prepared for each assay using a Comet Assay Electrophoresis System (Trevigen). The samples were then analyzed by fluorescence confocal microscopy using an Olympus FV1000. We analyzed tail moment using software provided by Trevigen.

### Xenograft mouse model

For the in vivo tumor formation assays, female BALB/c nude mice (4–5 weeks old) (Shanghai Lingchang Biology Co., LTD, China) were randomly divided into five groups that received inoculation of the following cells: Ishikawa-NC, Ishikawa-SOCS2-AS1, HEC-1A-NC, HEC-1A-shSOCS2-AS1, or HEC-1A-shSOCS2/shAURKA cells. In all, 2 × 10^6^ were suspended in 200 μl of serum-free DMEM and subcutaneously injected into the flank of each nude mouse. The tumor sizes and mouse weights were measured every 3 days, and tumor volumes were calculated as *V* (mm^3^) = width^2^ (mm^2^) × length (mm)/2. The mice were manipulated and housed according to protocols, and sacrificed after 24 days. All procedures were approved by the Animal Ethics Review Committee of Shanghai Jiaotong University School of Medicine.

### Statistical analysis

We performed all statistical analyses with SPSS 23.0, and results are presented as means ± standard deviation from at least three separate experiments. Student’s *t* test was conducted to analyze the significance of mean values between the two groups, whereas enumeration data were evaluated using *χ*^2^ test or Fisher exact-probability test. *P* < 0.05 was considered to be statistically significant.

## Results

### SOCS2-AS1 is downregulated in human EC and is negatively correlated with tumor progression

**Fig. 1:**
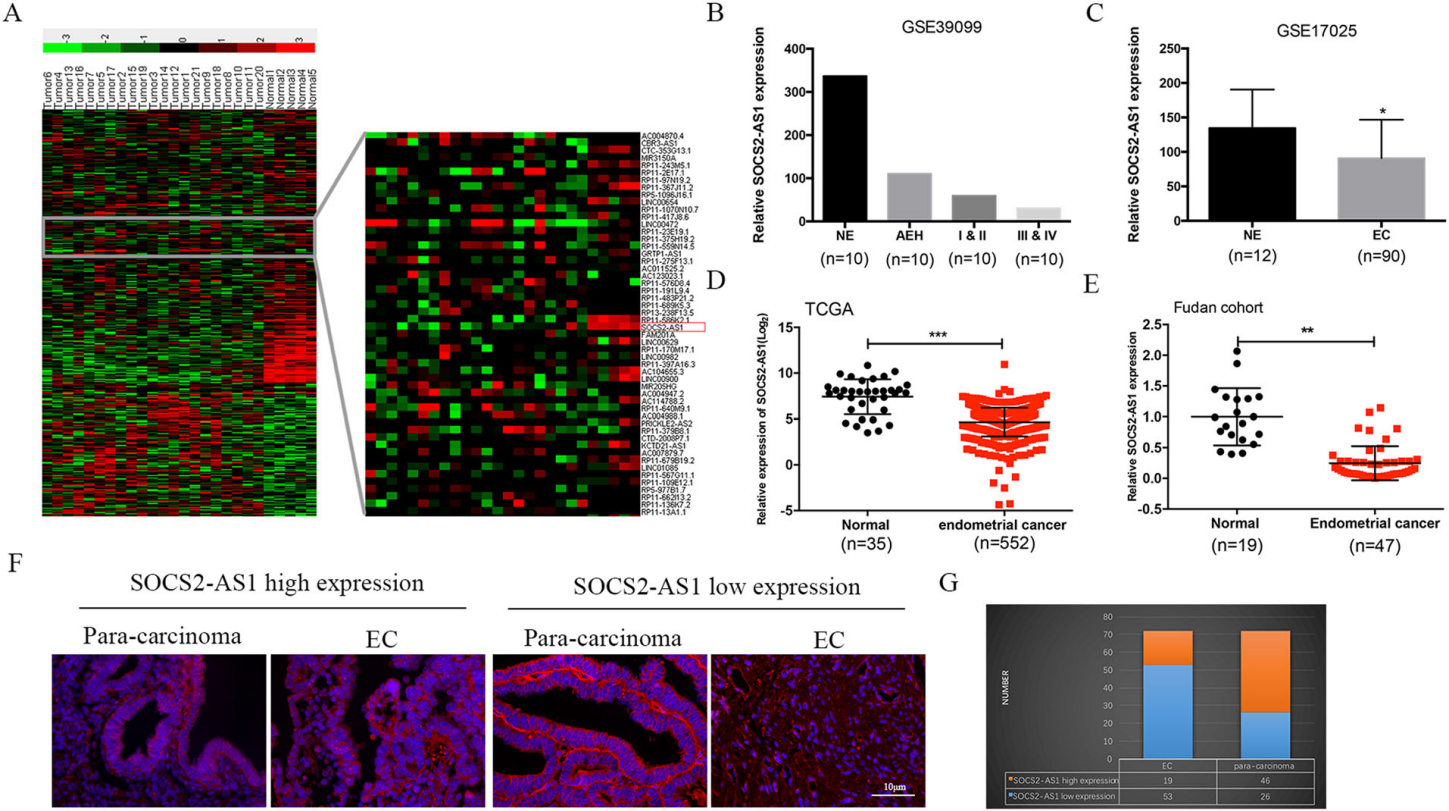
SOCS2-AS1 expression is diminished in EC tissues. **A** Heatmap showing the differential expression of lncRNAs in EC (*n* = 21) and NE tissues (*n* = 5), including the decreased levels of SOCS2-AS1. **B** Expression levels of SOCS2-AS1 in NE, AEH, stage I/II, and stage III/IV EC tissues (GSE39099). **C** SOCS2-AS1 expression is reduced in EC compared with NE tissues (GSE17025). **D** SOCS2-AS1 expression in EC compared with NE was analyzed using the TCGA database. **E** SOCS2-AS1 was detected in 47 EC tissues and 19 normal NE tissues by qPCR. **F** Representative images of FISH for SOCS2-AS1 in paracancerous and EC tissues detected on a tissue microarray. **G** Numbers of samples with high or low staining intensity for SOCS2-AS1 are shown. NE normal endometrium, AEH atypical endometrial hyperplasia.

To identify the functional lncRNAs involved in EC progression, we executed RNA-seq to profile lncRNA expression in a cohort of normal endometrial tissues (*n* = 5) and Ec tissues (*n* = 21). A total of 4114 lncRNAs were represented, of which 2791 were significantly upregulated (red) and 1323 were downregulated (green) when filtered using a threshold of a fold-change ≥2 and *P* < 0.05 (Fig. 1A). Among these differentially expressed lncRNAs, SOCS2-AS1 was found to be downregulated in nearly all EC tissues. In addition, analysis of two previously studied microarray data sets from the Gene Expression Omnibus (GEO) repository database also confirmed the loss of SOCS2-AS1 in EC (GEO datasets GSE39099 and GSE17025) (Fig. 1B, C). Consistent with our data, SOCS2-AS1 was decreased in the TCGA data set consisting of 552 patients with EC (Fig. 1D). To further verify the sequencing results, we quantified SOCS2-AS1 expression by qPCR in one independent cohort of 47 EC tissues and 19 normal tissues, and we found that SOCS2-AS1 was significantly downregulated in EC compared with normal endometrial tissues (Fig. 1E).

Next, we validated the downregulation of SOCS2-AS1 in cohort 2 composed of 72 paired EC tissues and adjacent normal tissues using FISH, and observed that the proportion of low-expressed SOCS2-AS1 in EC samples was significantly higher than that in paracarcinoma samples (49/72 vs. 26/72 [68.1 vs. 36.1%], respectively) (Fig. 1F, G; *P* < 0.05). We then analyzed the relationship between SOCS2-AS1 expression and EC progression, and noted that SOCS2-AS1 was negatively correlated with prognostic clinical factors that included clinical stage and Ki67 level (Table 1). These findings demonstrated that loss of SOCS2-AS1 was a critical event in endometrial carcinogenesis, and that this characteristic could be used as a prognostic indicator for EC patients.

**Table 1:** The association of SOCS2-AS1 expression in 72 endometrial cancer patients with clinicopathologic characteristics.

		SOCS2-AS1			
Parameters	Total	Low (no.)	High (no.)	*χ*^2^	*P* value
Age (years)				1.131	0.288
<50	61	40	21		
≥50	11	9	2		
Histology grade				0.762	0.383
G1+G2	62	41	21		
G3	10	8	2		
Clinical stage				4.917	**0.027**
I+II	58	36	22		
III+IV	14	13	1		
Ki67 staining				5.133	**0.023**
<50	46	27	19		
≥50	26	22	4		
Pathologic type				0.353	0.553
Endometrioid	67	45	22		
Non-endometrioid	5	4	1		
Depth of myometrial invasion				1.647	0.199
<1/2	56	36	20		
≥1/2	16	13	3		
Lymph node metastasis				0.006	0.937
No	43	33	10		
Yes	4	3	1		

Items with statistical differences are shown in bold.

### SOCS2-AS1 suppresses EC cell growth both in vitro and in vivo

**Fig. 2:**
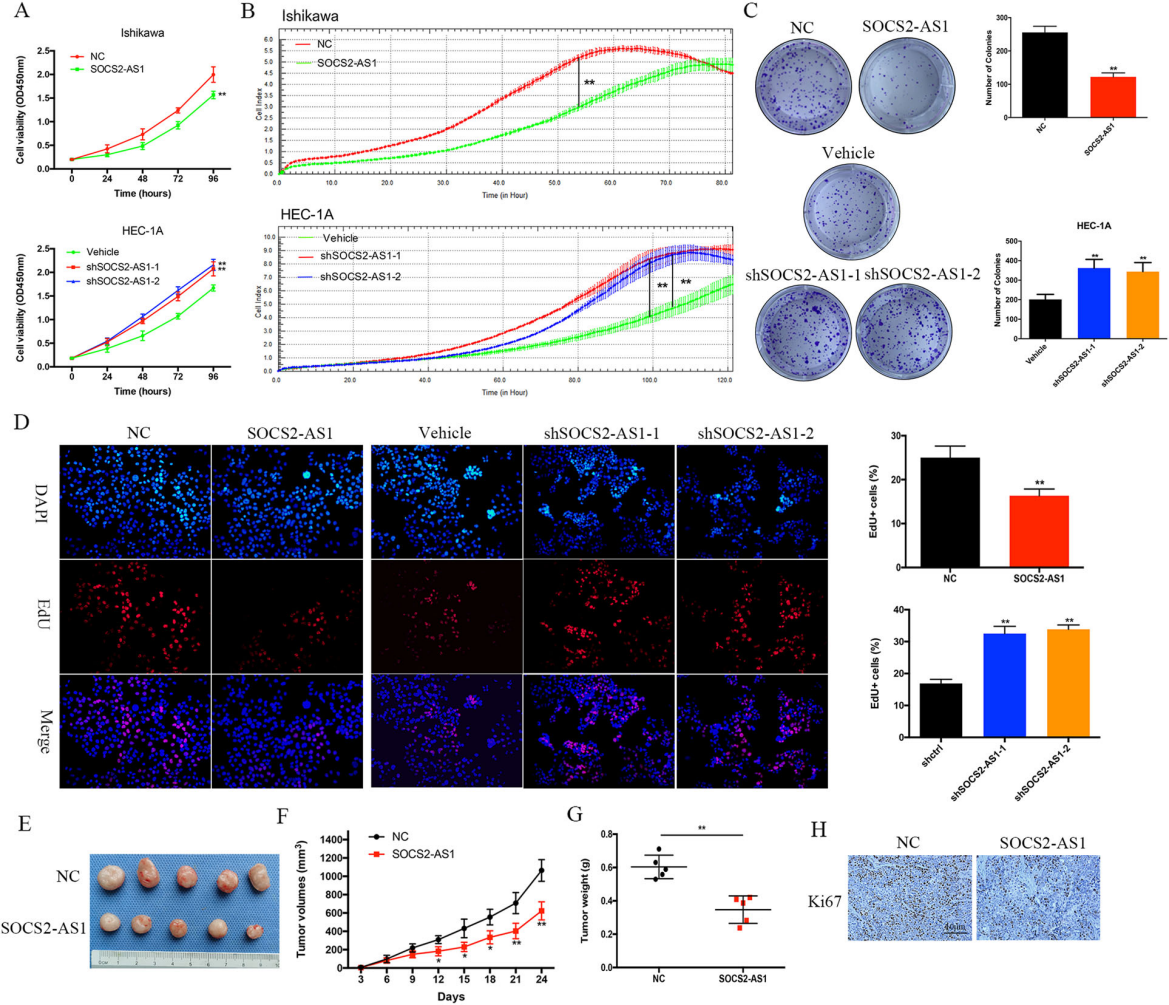
SOCS2-AS1 plays a tumor-suppressing role in EC. SOCS2-AS1 overexpression inhibited while SOCS2-AS1-knockdown promoted EC cell proliferation as examined using the CCK8 assay **A**, RTCA assay **B**, colony-formation assays **C**, and EdU assay **D**. **E**–**H** SOCS2-AS1 overexpression inhibited Ishikawa cell xenograft growth. SOCS2-AS1-overexpressing Ishikawa cells or control Ishikawa cells were subcutaneously grafted into nude mice. At the end of the experiments, mice bearing tumors were killed, and the tumor tissues were collected, photographed **E**, and weighed **G**. **F** Xenograft growth curves. **H** IHC staining for Ki67 in the two indicated groups of mouse tumor specimens.

We first assessed the abundance of SOCS2-AS1 in six common EC cell lines by qPCR (Supplementary Fig. 1A), and we selected Ishikawa cells with the lowest SOCS2-AS1 and HEC-1A cells with the highest SOCS2-AS1 levels for further analysis. To directly test the putative tumor-suppressive functions of SOCS2-AS1, we used lentiviral vectors to stably overexpress human SOCS2-AS1 in the Ishikawa cell line and to stably knockdown SOCS2-AS1 in HEC-1A using two different shRNA sequences (Supplementary Fig. 1B, C). CCK8 and RTCA assays showed that overexpressed SOCS2-AS1 significantly inhibited cellular proliferation, whereas, in contrast, knockdown of SOCS2-AS1 promoted proliferation (Fig. 2A, B). The results of the colony-formation assay revealed that clonogenic ability was also significantly attenuated following SOCS2-AS1 overexpression and knockdown of SOCS2-AS1 augmented EC cell-colony formation (Fig. 2C). Similarly, the 5-ethynyl-2’-deoxyuridine (EdU) proliferation assays indicated that cell replication was significantly suppressed upon SOCS2-AS1 overexpression (Fig. 2D).

The effects of SOCS2-AS1 dysregulation on tumor growth were further tested in vivo by injecting Ishikawa vector cells and SOCS2-AS1-overexpressing cells into the right flanks of nude mice. The tumor sizes were measured every 3 days, and the results showed that tumor growth was inhibited by SOCS2-AS1 overexpression (Fig. 2F). At 24 days, tumors were dissected, photographed, and weighed. Consistent with our in vitro results, tumors generated by SOCS2-AS1-overexpressing cells were significantly smaller and lighter than the tumors from control cells (Fig. 2E and G); additionally, cellular proliferation was also diminished in the tumors from the former cells as determined by Ki67 staining (Fig. 2H). Collectively, these in vitro and in vivo observations indicated that SOCS2-AS1 acts as a tumor suppressor in the development of EC.

### SOCS2-AS1 accelerates cellular apoptosis and inhibits cell-cycle progression in EC cell lines

**Fig. 3:**
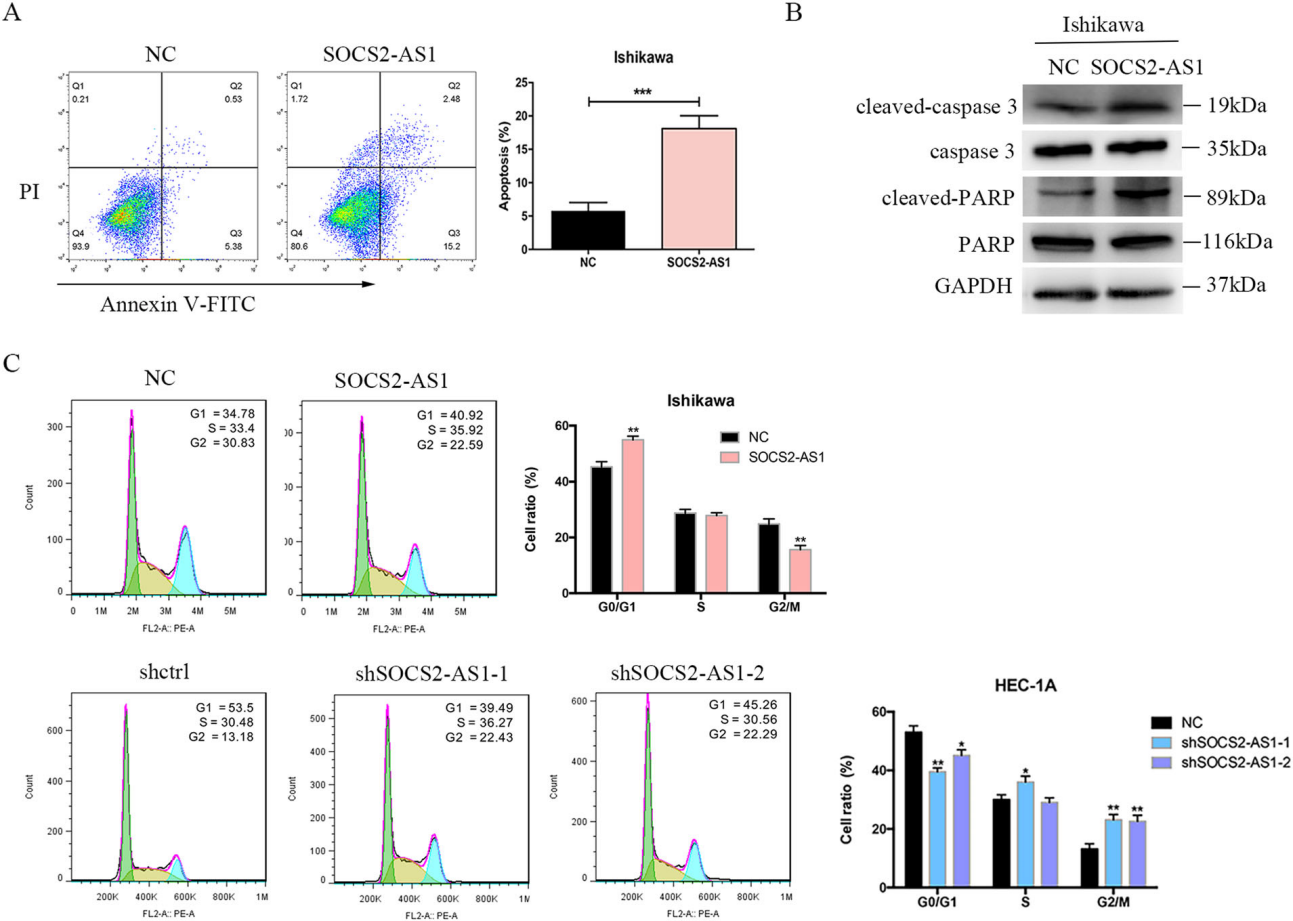
Effect of SOCS2-AS1 on cell cycle and apoptosis. **A** Apoptotic rates of cells were analyzed with flow cytometry. **B** Western blotting analysis of apoptotic protein after SOCS2-AS1 overexpression in Ishikawa cell lines. **C** The percentage of cells in each phase of the cell cycle was determined by flow cytometry. The bar chart represents the percentage of cells in G0/G1, S, or G2/M phase, as indicated (right). *P < 0.05, **P < 0.01.

To investigate the mechanism by which SOCS2-AS1 inhibits cell growth, we examined the effect of SOCS2-AS1 on cellular apoptosis by flow cytometry. Ectopic SOCS2-AS1 expression enhanced both early and late apoptosis in Ishikawa cells (Fig. 3A). Induction of apoptosis by SOCS2-AS1 was also evident by the increased protein expression of cleaved forms of caspase-3 and PARP in stably transfected Ishikawa cells relative to controls (Fig. 3B). Moreover, when we analyzed the effect of SOCS2-AS1 on cell-cycle progression, we observed that compared with control cells, upregulated SOCS2-AS1 expression induced cell-cycle arrest in Ishikawa cells lines, as significantly more cells were in G1/G0 phase, whereas SOCS2-AS1 silencing produced the opposite effect (Fig. 3C). Therefore, the antitumor effect of SOCS2-AS1 arises through suppression of cell-cycle progression and promotion of cell apoptosis in EC cells.

### SOCS2-AS1 is predominantly located in the cytoplasm and binds to the AURKA

**Fig. 4:**
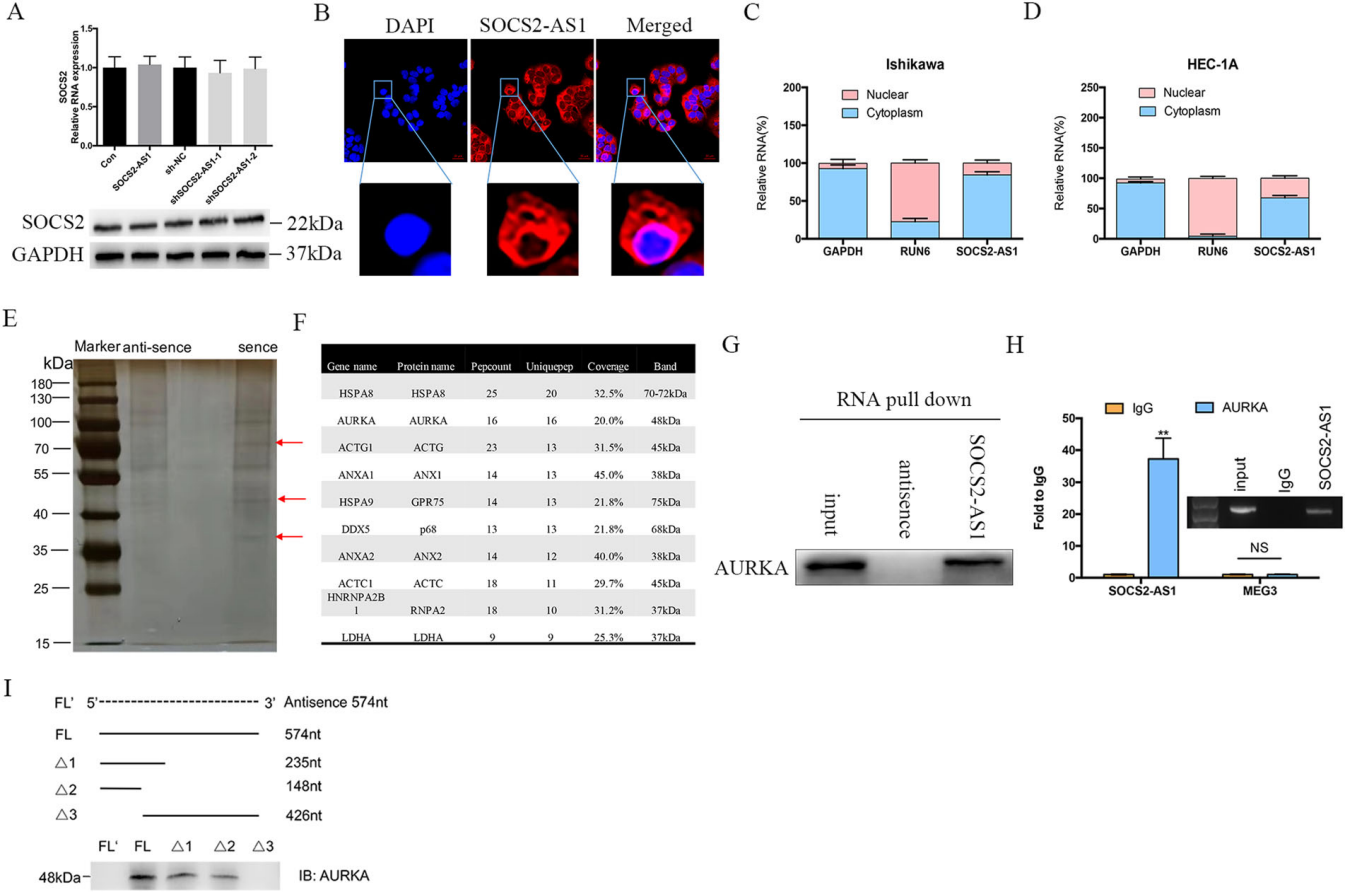
SOCS2-AS1 forms a complex with AURKA. **A** The mRNA and protein levels for SOCS2 after SOCS2-AS1 overexpression or knockdown. **B** RNA FISH in HEC-1A cells showed that SOCS2-AS1 was principally located in the cytoplasm. **C**, **D** Subcellular localization of SOCS2-AS1 was analyzed by qPCR in cellular fractions of HEC-1A and Ishikawa cells. GAPDH was used as a cytosolic marker, and U6 was used as a nuclear marker. **E** Silver staining of biotinylated SOCS2-AS1-associated proteins. Three SOCS2-AS1-specific bands (red arrows) were excised and analyzed using mass spectrometry. **F** Top-10 protein hits of bands, resulting from MS are labeled with a red arrow in **E**. **G** Western blot of the AURKA proteins from sense and antisense SOCS2-AS1 pull-down assays. **H** RNA immunoprecipitation experiments were performed using anti-AURKA antibody, and qPCR was used to detect SOCS2-AS1. MEG3 was used as a negative control. **I** Western blot of AURKA in samples pulled down by full-length (FL) or truncated SOCS2-AS1 (Δ1, 1–235; Δ2, 1–148; Δ3, 149–574).

To elucidate the molecular mechanism governing SOCS2-AS1 action, we first examined whether it acts in a *cis* fashion, affecting neighboring gene expression. qPCR and western blots, however, showed that overexpression and knockdown of SOCS2-AS1 exerted no effect on the expression of SOCS2 (Fig. 4A), indicating that it may act *trans*. FISH assays in HEC-1A cells showed that SOCS2-AS1 exhibited a predominantly cytoplasmic localization (Fig. 4B), which was also confirmed by nuclear/cytoplasmic fractionation assays (Fig. 4C). We then performed an RNA pull-down assay followed by a proteomic analysis of the SOCS2-AS1-associated protein complex in HEC-1A cells (Fig. 4E). Among the top-10 specific interactors with sense SOCS2-AS1 (Fig. 4F), we were most interested in 1 protein, AURKA, which is reported to occupy an important role in the cell cycle, genomic integrity, and EC progression^17^. To further corroborate the interaction between SOCS2-AS1 and AURKA, we performed the RNA pull-down assay followed by western blotting using AURKA antibodies. Our results showed that labeled SOCS2-AS1 RNA—but not an antisense SOCS2-AS1—specifically retrieved AURKA from HEC-1A cell extracts (Fig. 4G). This interaction between SOCS2-AS1 and AURKA was also confirmed by RIP assays in HEC-1A cells (Fig. 4H). To identify the AURKA-interacting region of SOCS2-AS1, we constructed and biotinylated five fragments of SOCS2-AS1 (FL, full-length SOCS2-AS1; FL’, full-length antisense SOCS2-AS1; Δ1, 1–235 bp; Δ2, 1–148 bp; and Δ3, 236–426 bp), and used them in the pull-down assay with HEC-1A cell lysates. In addition, we found that the 5′ fragment of SOCS2-AS1 mediated the interaction with AURKA (Fig. 4I). Thus, the AURKA protein is a specific interactive partner of the SOCS2-AS1 in EC.

### SOCS2-AS1 decreases the stability of AURKA by promoting ubiquitin/proteasome-dependent degradation

**Fig. 5:**
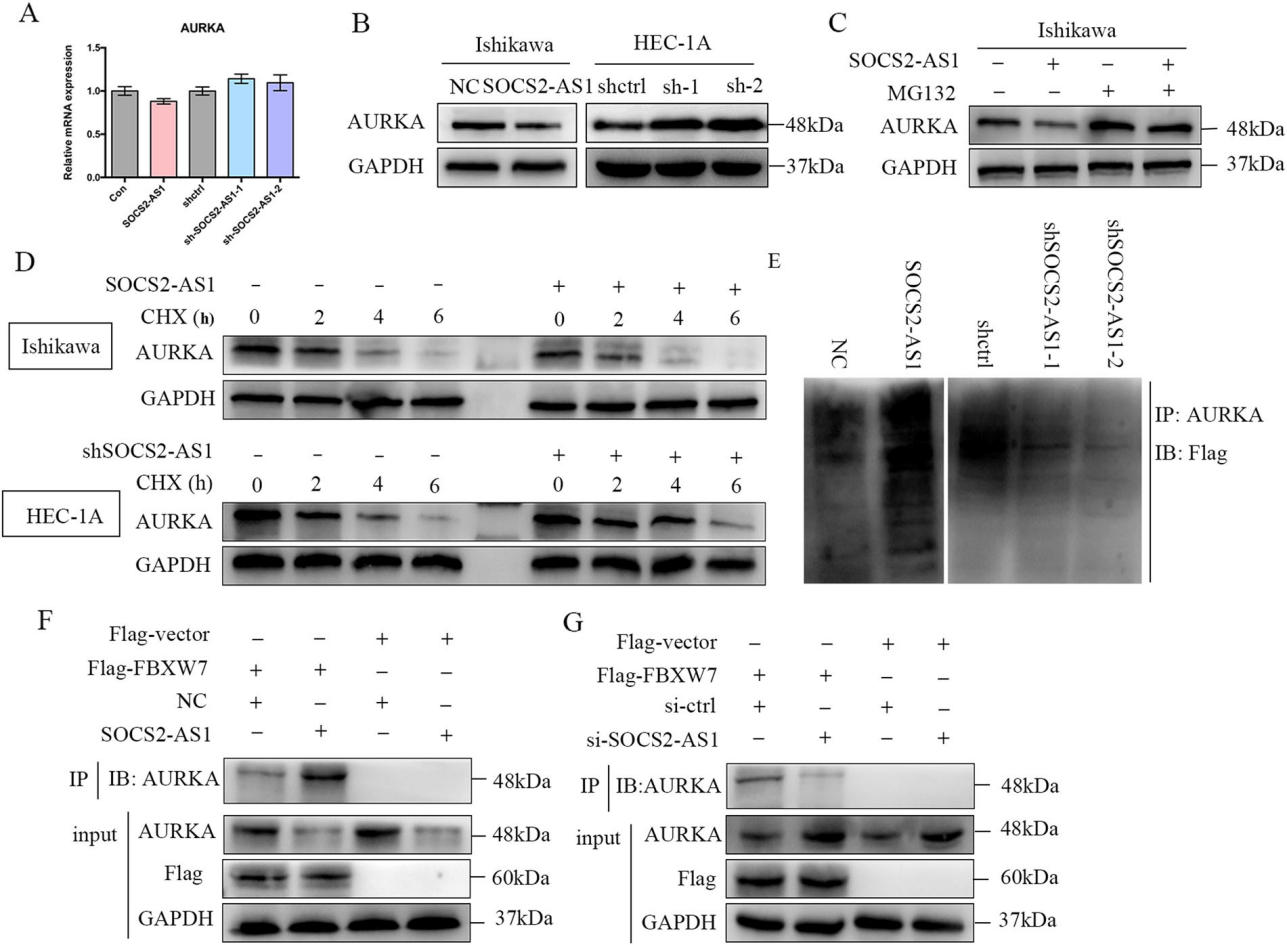
SOCS2-AS1 regulates AURKA protein stability via ubiquitination and proteasomal degradation. **A** AURKA mRNA was determined by qPCR after SOCS2-AS1 overexpression in Ishikawa cells or knockdown in HEC-1A cells. **B** Immunoblotting for AURKA protein levels after SOCS2-AS1 overexpression or knockdown. GAPDH served as the internal control. **C** Western blot images of AURKA expression in Ishikawa cells expressing control or SOCS2-AS1 after treatment with DMSO or MG132. **D** Ishikawa cells with or without SOCS2-AS1 overexpression and HEC-1A cells with or without SOCS2-AS1 knockdown were treated with 25 μg/mL of cycloheximide (CHX) for the indicated time. The whole-cell lysate was detected by western blotting analysis. **E** Western blot images showing AURKA-associated ubiquitination in SOCS2-AS1-overexpressing cells and after the knockdown. **F** Immunoprecipitation to detect the association between AURKA and FBXW7 after SOCS2-AS1 overexpression in Ishikawa cells. **G** Immunoprecipitation to detect the association between AURKA and FBXW7 after SOCS2-AS1 knockdown in HEC-1A cells.

To explore the mechanism underlying the association between SOCS2-AS1 and AURKA, we tested whether SOCS2-AS1 affected AURKA expression, and observed that SOCS2-AS1 manifested no significant effect on AURKA mRNA levels (Fig. 5A). However, AURKA protein levels were dramatically increased with SOCS2-AS1 silencing, and were reduced with overexpression of SOCS2-AS1 (Fig. 5B). The degradation of endogenous AURKA in cells overexpressing SOCS2-AS1 was blocked by MG132 (Fig. 5C) (a proteasome inhibitor), suggesting that SOCS2-AS1 is involved in the post-translational regulation of AURKA. Moreover, following treatment with the protein synthesis inhibitor cycloheximide, SOCS2-AS1 knockdown increased AURKA protein half-life, whereas SOCS2-AS1 activation decreased the half-life of the AURKA protein in EC cells (Fig. 5D). Furthermore, the ubiquitination levels of AURKA significantly increased in the SOCS2-AS1-overexpressing cells, whereas the ubiquitination levels of AURKA decreased in the cells that underwent SOCS2-AS1 knockdown (Fig. 5E). Previous reports identified FBXW7 as an E3 ligase for AURKA ubiquitination and degradation^18^, and this prompted us to assess the function of SOCS2-AS1 in the interaction between FBXW7 and AURKA in EC cells. Indeed, SOCS2-AS1 overexpression significantly enhanced the association between FBXW7 and AURKA (Fig. 5F), whereas SOCS2-AS1 knockdown markedly decreased this association in EC cells (Fig. 5F). Collectively, these results indicated that SOCS2-AS1 decreases the stability of AURKA via the promotion of its ubiquitin/proteasome-dependent degradation.

### A large set of genes involved in cell cycle, DNA replication, and repair is regulated by SOCS2-AS1

**Fig. 6:**
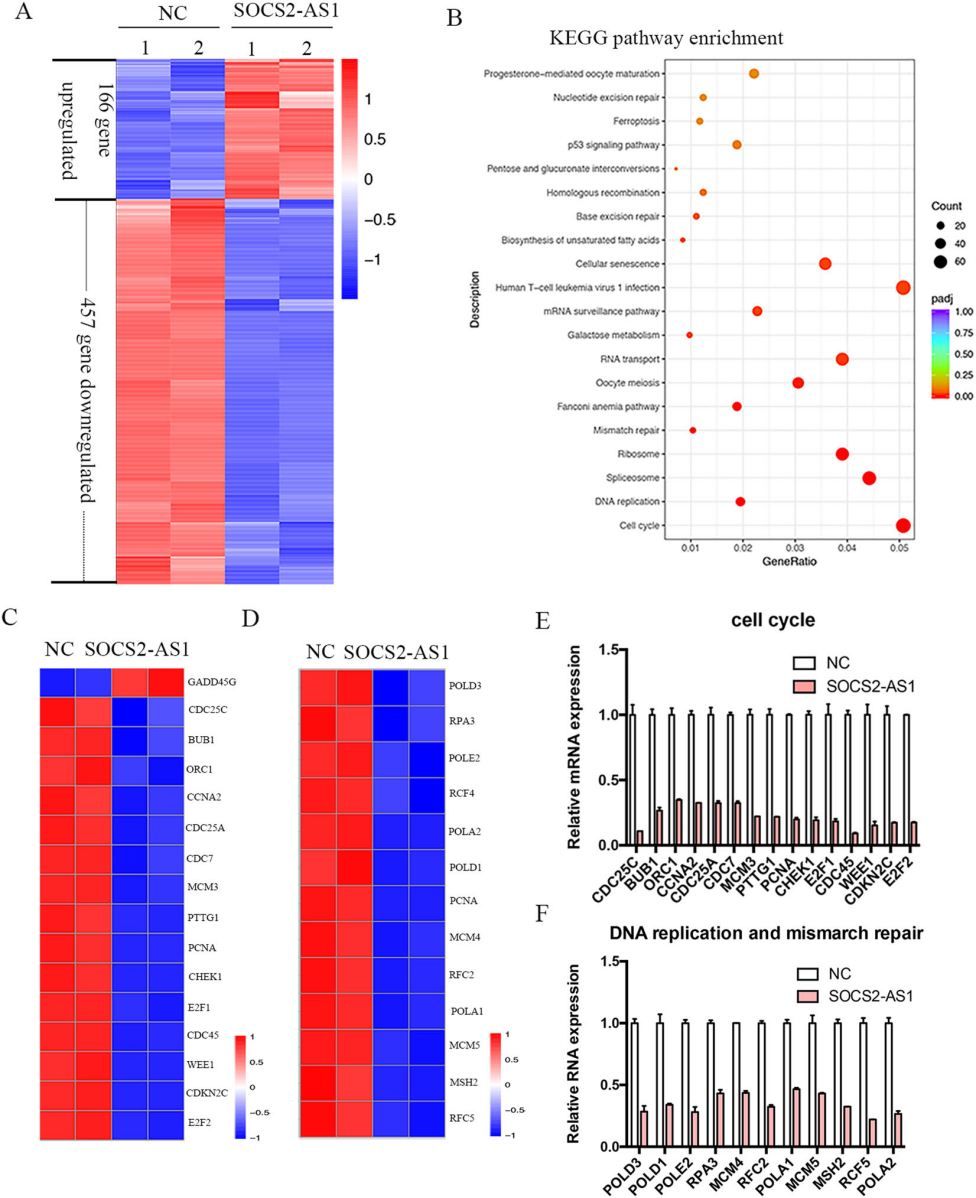
Effect of SOCS2-AS1 overexpression on the transcriptional landscape of Ishikawa cells. **A** Heatmap showing differential transcripts between normal and SOCS2-AS1-overexpressing groups. **B** KEGG pathway analysis of the differential genes regulated by SOCS2-AS1. **C**, **D** Cell cycle and DNA repair signaling-related genes in the SOCS2-AS1-overexpressing Ishikawa cell transcriptome. **E**, **F** qPCR validation of representative genes involved in the cell cycle, DNA replication, and mismatch repair after AURKA overexpression. Data shown represent the means (±SD) of biologic triplicates.

To gain insight into the molecular function of SOCS2-AS1, we profiled Ishikawa cell gene expression after SOCS2-AS1 overexpression using RNA-seq. Differential expression analysis revealed that 623 genes were disrupted by SOCS2-AS1, of which a majority (73%, 457/623) were downregulated, whereas only 166 genes were upregulated (Fig. 6A). KEGG pathway analysis revealed that the genes regulated by SOCS2-AS1 were mainly enriched in pathways associated with cell cycle, DNA replication, and mismatch repair (Fig. 6B, C). We then selected several SOCS2-AS1-repressed genes that have been reported to be involved in cancer progression, including key cancer genes, such as CHEK1, PTTG1, MSH2, CDC25C, PNCA, WEE1, and etc, and validated their expression by qPCR. Consistent with the Seq analysis, our results confirmed that the levels of these genes were all modestly decreased after SOCS2-AS1 overexpression (Fig. 6D). Therefore, we hypothesized that the deregulation of these cell growth-associated genes was directly responsible for the decreased cell growth observed in the SOCS2-AS1-overexpressing cells.

### Inhibition of AURKA reverses the cell growth that occurs due to loss of SOCS2-AS1 in EC Cells

**Fig. 7:**
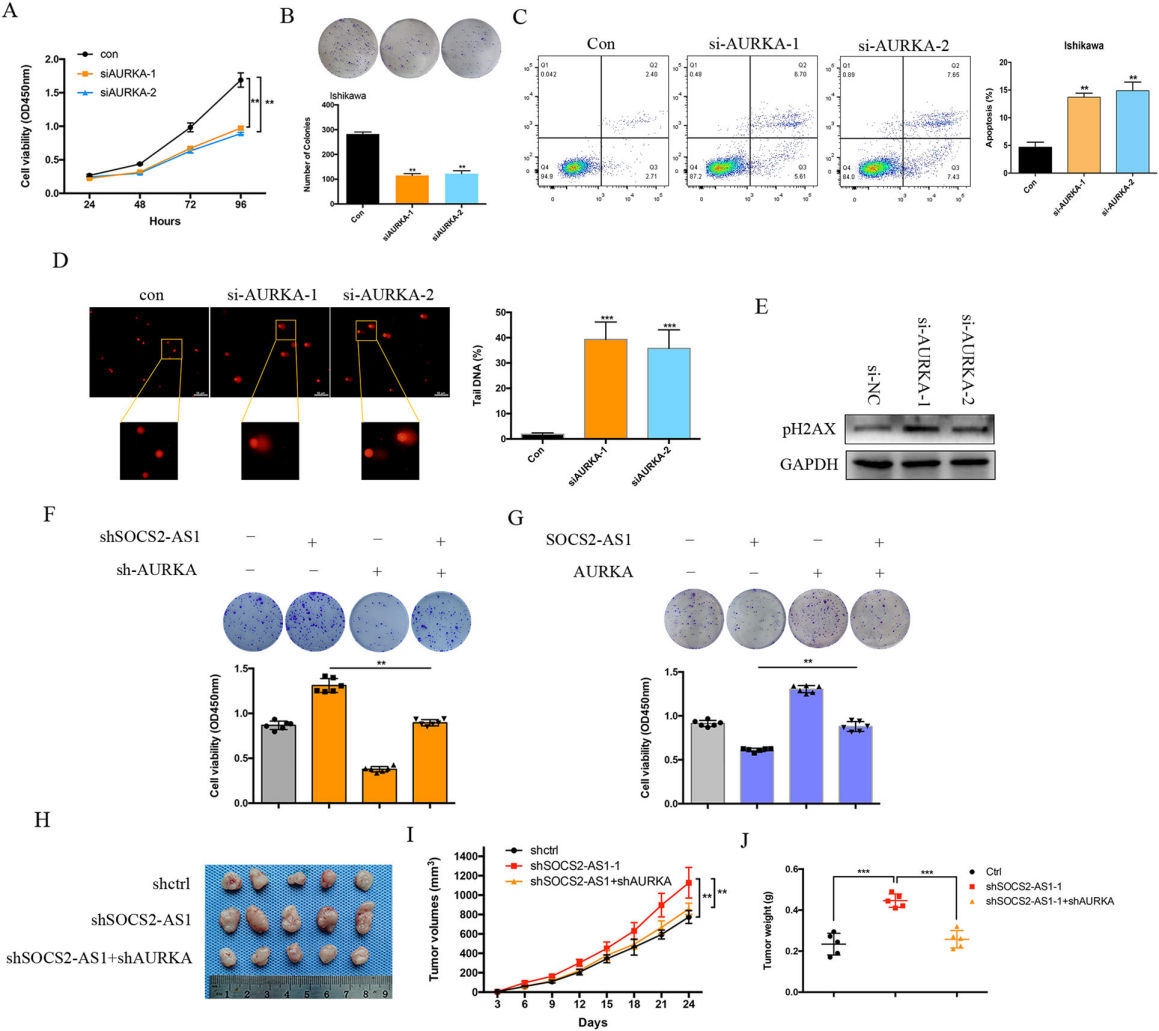
Inhibition of AURKA reverses cellular growth induced by SOCS2-AS1 loss. **A** CCK8 assay was performed in Ishikawa cells transfected with control or AURKA siRNA plasmid. **B** Colony-formation assays of Ishikawa cells transfected with control or AURKA siRNA plasmid. **C** Apoptotic rates of cells were analyzed in Ishikawa cells with control or AURKA siRNA plasmid. **D** Comet assays used to measure DNA damage in Ishikawa transfected with AURKA siRNA plasmid. **E** Immunoblotting of pH2AX in Ishikawa cells transfected with AURKA siRNA plasmid. **F** AURKA and SOCS2-AS1 double-knockdown rescue SOCS2-AS1 loss-mediated EC cell growth. CCK8 and colony-formation assays were measured in HEC-1A cells transfected with control, AURKA siRNA, SOCS2-AS1 siRNA, or co-transfected with both. **G** AURKA could reverse SOCS2-AS1-mediated EC cell growth inhibition. CCK8 and colony-formation assays were measured in Ishikawa cells transfected with control, AURKA, SOCS2-AS1, or co-transfected with both. **H** HEC-1A xenograft upon SOCS2-AS1 knockdown with or without knockdown of AURKA. **I** Growth of HEC-1A xenograft tumors was measured using tumor volume, and tumor size was monitored every 3 days. Data (*n* = 5) were analyzed using a paired-sample Student’s *t* test. **J** Tumor weight was measured in each group at the end of the experiment.

To further explore the clinical implications of AURKA in EC, we silenced AURKA in EC Ishikawa cells using siRNA. Notably, AURKA silencing dramatically impaired cellular proliferation and colony formation, and enhanced cellular apoptosis in EC cell lines (Fig. 7A–C). Since previous studies have shown that AURKA is involved in genomic integrity, we next examined the role of AURKA knockdown with respect to DNA damage. DNA double-strand breaks (DSB) constitute a severe form of DNA damage that can be detected with the neutral comet assay. Our comet assays in EC cell lines showed significantly increased amounts of DNA in the tails, indicating increased DNA damage induced by AURKA depletion (Fig. 7D). Phosphorylation of histone H2AX (a marker of DSB damage and repair) was also increased by AURKA silencing in EC cell lines (Fig. 7E). To determine whether AURKA mediated the biologic functions of SOCS2-AS1 in EC, we employed rescue assays. Strikingly, the silencing of AURKA almost completely abolished the induction of cell growth elicited by SOCS2-AS1 depletion, both in vivo and vitro (Fig. 7F, H–J). Although overexpression of AURKA could reverse the inhibitory effect of SOCS2-AS1 on cell growth (Fig. 7G). These results suggest that AURKA is a critical mediator of the SOCS2-AS1 phenotype.

## Discussion

Although lncRNAs are implicated in the initiation and development of various tumors, the potential involvement of lncRNA(s) is poorly defined for human EC. In the present study, we identified by RNA-sequencing analysis that SOCS2-AS1 was markedly downregulated in EC. We further validated the SOCS2-AS1 expression pattern in clinical EC samples in another cohort (TCGA) and in two other GEO data sets. The low expression of SOCS2-AS1 we observed for EC patients was also negatively correlated with clinical stage and Ki67 level. These data imply that SOCS2-AS1 participates in crucial functions in EC.

SOCS2-AS1 is a poorly defined lncRNA. Zheng’s work showed that SOCS2-AS1 was decreased in colorectal cancer (CRC) and that its downregulation predicted a poor prognosis in patients with CRC^19^. However, studies on prostate cancer indicated a discordant oncogenic role and different molecular mechanisms underlying SOCS2-AS1 action^20^, thereby suggesting tissue-specific regulation of SOCS2-AS1 expression. Nonetheless, the significance of SOCS2-AS1 in EC progression is currently unappreciated. We further investigated the functional roles and mechanisms subserving SOCS2-AS1 using gain- and loss-of-function assays in vitro and in vivo, and demonstrated that in EC cell lines and xenograft mouse models, SOCS2-AS1 overexpression resulted in a significant decrease in cell growth and tumorigenicity, whereas SOCS2-AS1 knockdown accelerated cell growth.

LncRNAs can act in combination with specific proteins to exert different functions according to subcellular location. For example, in the nucleus, lncRNAs can function as guides to regulate target specificity for transcription factors or epigenetic regulators, and to modulate chromosomal instability^21,22^. In addition, cytoplasmic lncRNAs may participate in regulating protein stability and modification^23,24^. In the present study, our FISH and RNA fractionation experiments showed that SOCS2-AS1 was located predominantly in the cytoplasm. Our RNA-binding protein pull-down and mass spectrometry analyses also identified AURKA as one of the few proteins bound to SOCS2-AS1. Furthermore, we established an interaction between SOCS2-AS1 and AURKA in EC cells using RNA pull-down and RIP assays, and found that SOCS2-AS1 lies upstream of AURKA and regulates its stability. SOCS2-AS1 appears to promote AURKA degradation by reinforcing the interaction between AURKA and FBXW7, an E3 ubiquitin ligase. Functional analyses and a mouse xenograft-tumor model also showed that knockdown of SOCS2-AS1 markedly induced EC cell proliferation and that this effect was completely rescued by simultaneous knockdown of both SOCS2-AS1 and AURKA. Using RNA-seq we found that SOCS2-AS1 affected key cell-cycle and DNA damage-related genes such as CDC25C, WEE1, CHEK1, and MCM2. Thus, we showed that the impact of SOCS2-AS1 overexpression was caused by a mixed phenotype that included a decrease in proliferation and induction of apoptosis.

AURKA is located at chromosome 20q13.2 and shown to play an important role during mitosis^25^, as AURKA regulates the cell-cycle checkpoint and maintains genomic integrity. Several studies have shown that AURKA is frequently amplified in several tumors, including breast, pancreatic, colorectal, gastric, and ovarian carcinomas, and in some cases is associated with a poor prognosis^26–28^. A few specific AURKA inhibitors have now moved forward in clinical trials, including MLN8237 (clinical trial #NCT01482962) and ENMD-2076 (#NCT01914510, #NCT01639248, and #NCT01104675). Patients with EC, however, lack effectively targeted therapies. Although some therapeutic targets such as immune-checkpoint inhibitors (targeting programmed cell death protein 1/programmed cell death protein ligand 1), HER2 inhibitors, and antiangiogenic agents have been uncovered^29^, their efficacy in treating patients with EC remains unclear. Identification of biomarkers that can be used to reliably predict benefits of AURKA inhibitors should therefore be developed as an integral component of clinical trials in the pursuit of personalized medicine for EC. Finally, we provided in vitro and in vivo evidence that the growth of cells and tumors that lack SOCS2-AS1 may be effectively blocked by AURKA inhibition, raising the possibility that SOCS2-AS1 loss in cancer cells may predict an anti-AURKA benefit.

## Conclusions

In conclusion, we characterized the lncRNA SOCS2-AS1 as a tumor suppressor in EC. SOCS2-AS1 exerted its tumor-suppressive activity through interaction with and promotion of AURKA ubiquitin degradation, thereby reducing the levels of AURKA protein and its target mRNAs—including those mRNAs directly associated with cancer cell growth. SOCS2-AS1 may therefore serve as a prognostic predictor for patients with EC, and the SOCS2-AS1-AURKA axis remains a potential therapeutic target for EC treatment.

## Supplementary information

supplementary

supplementary table 1
