# Supplementary material for: The long-noncoding RNA SOCS2-AS1 suppresses endometrial cancer progression by regulating AURKA degradation
Source: Cell Death Dis. 2021 Apr 6;12(4):351. doi: 10.1038/s41419-021-03595-x (PMC8024384; doi:10.1038/s41419-021-03595-x)
Supplement: Supplementary file 1 — supplementary [file 41419_2021_3595_MOESM1_ESM.docx]

**Supplement Figure legend**

**Figure S1**

1. SOCS2-AS1 levels measured by qPCR in various EC cell lines.
2. Confirmation of SOCS2-AS1 overexpression by qPCR in Ishikawa cells.
3. Confirmation of SOCS2-AS1 knockdown by qPCR in HEC-1A cells.
